# Computational Fitness Landscape for All Gene-Order Permutations of an RNA Virus

**DOI:** 10.1371/journal.pcbi.1000283

**Published:** 2009-02-06

**Authors:** Kwang-il Lim, John Yin

**Affiliations:** Department of Chemical and Biological Engineering, University of Wisconsin Madison, Madison, Wisconsin, United States of America; University of Texas at Austin, United States of America

## Abstract

How does the growth of a virus depend on the linear arrangement of genes in its genome? Answering this question may enhance our basic understanding of virus evolution and advance applications of viruses as live attenuated vaccines, gene-therapy vectors, or anti-tumor therapeutics. We used a mathematical model for vesicular stomatitis virus (VSV), a prototype RNA virus that encodes five genes (N-P-M-G-L), to simulate the intracellular growth of all 120 possible gene-order variants. Simulated yields of virus infection varied by 6,000-fold and were found to be most sensitive to gene-order permutations that increased levels of the L gene transcript or reduced levels of the N gene transcript, the lowest and highest expressed genes of the wild-type virus, respectively. Effects of gene order on virus growth also depended upon the host-cell environment, reflecting different resources for protein synthesis and different cell susceptibilities to infection. Moreover, by computationally deleting intergenic attenuations, which define a key mechanism of transcriptional regulation in VSV, the variation in growth associated with the 120 gene-order variants was drastically narrowed from 6,000- to 20-fold, and many variants produced higher progeny yields than wild-type. These results suggest that regulation by intergenic attenuation preceded or co-evolved with the fixation of the wild type gene order in the evolution of VSV. In summary, our models have begun to reveal how gene functions, gene regulation, and genomic organization of viruses interact with their host environments to define processes of viral growth and evolution.

## Author Summary

Although many viruses are linked to diseases that adversely impact the health of their human, animal, and plant hosts, viruses could help promote wellness and treat disease if their “good traits” could be harnessed. Potentially useful virus traits include their abilities to stimulate a robust immune response, target specific tissues for the delivery of foreign genes, and destroy tumors. The exploitation of such traits in the engineering of virus-based vaccines, gene therapies and anti-cancer strategies is limited in part by our inability to control how viruses grow. Generally, viruses that grow poorly will be more desirable for vaccine applications, whereas viruses that grow and spread rapidly will be useful for destroying tumors. Further, gene therapies will rely on controlling the extent to which a therapeutic gene is delivered and expressed. Robust methods for controlling virus growth have yet to be discovered. However, for some viruses, such as vesicular stomatitis virus (VSV), growth can be very sensitive to the specific linear order of its five genes. Our current work is significant in combining experiments and computational models to identify which virus genes and genome positions most sensitively impact VSV growth, providing a foundation for its applications in human health.

## Introduction

**Figure 1:**
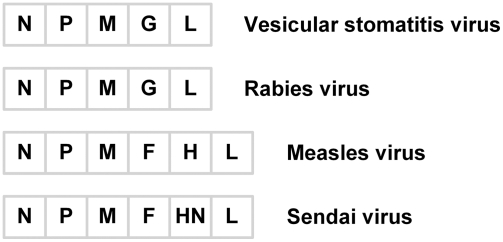
Different RNA viruses share a similar genome organization. These viruses carry a negative-sense single-stranded RNA genome.

The gene orders in the genomes of individual negative-sense single-stranded RNA viruses have been conserved [1]–[3]. More specifically, most viruses in the order *Mononegavirales*, abbreviated here as (–)ssRNA viruses, share a similar genome organization: 3′-*cap*-*phos*-*mat*-*env*-*pol*-5′, where *cap* encodes nucleocapsid protein (N), *phos* encodes phosphoprotein (P), *mat* encodes matrix protein (M), *env* or multiple analogous genes encode envelope protein(s) (G) or attachment (H and HN) and fusion proteins (F), and *pol* encodes polymerase protein (L) (Figure 1) [1],[2]. It has long been hypothesized that such gene-order conservation and similarity either reflect the absence of a genome recombination mechanism for this virus family [4] or arise from relevant fitness benefits. However, such a hypothesis has been recently challenged by several studies of (–)ssRNA viruses. First, a phylogenetic analysis of nucleoprotein and glycoprotein gene sequences of ebolaviruses from natural isolates suggested that recombination between different groups of ebolaviruses had occurred [5]. Another phylogenetic analysis of several genes of Hantaan virus, Mumps virus and Newcastle disease virus also strongly suggested that recombination in (–)ssRNA viruses could take place at low rates [6]. In addition, inverted gene orders of Pneumoviruses with similarities in protein and mRNA sequences (Turkey rhinotracheitis virus (TRTV): 3′-F-M2-SH-G-5′, respiratory syncytial virus (RSV) and pneumonia virus of mice (PVM): 3′-SH-G-F-M2-5′, avian pneumovirus (APV): 3′-F-M2-SH-G-5′) suggest the possibility for recombination events during their evolution [7]–[9]. Second, changes of gene orders in viral genomes have increased replication rates of some (–)ssRNA viruses. For example, when F and G genes were moved into promoter-proximal positions, replication rates of RSV mutants were increased up to 10-fold relative to wild type [10]. In addition, shuffling the P, M, and G genes of vesicular stomatitis virus (VSV) created mutants that could grow as well or better than wild type [11]. What is then the origin of gene orders in (–)ssRNA virus genomes? If a recombination mechanism was not available, how might the present specific gene orders have been selected from numerous possibilities? If genome recombination was possible, do the gene orders of current wild-type viruses represent those with the highest fitness? Answers to these questions could shed light on how RNA viruses have evolved, but they remain challenging to address.

To obtain some initial clues, we sought to compare the fitness of all possible gene-shuffled variants of a prototype (–)ssRNA virus based on predictions of their growth dynamics. By using mathematical expressions to account for the dynamics of gene expression and known interactions among gene products one may represent the development of virus growth with a mechanistic model of moderate, but not overwhelming complexity. Growth models of viruses aim to account for the synthesis, interactions and degradation of viral intermediates toward progeny production as they utilize the resources of host cells [12]–[14]. Such models can show how genome-wide regulation of viral gene expression can contribute to the integrated development of virus progeny. Previous work has shown how relocation of the gene encoding the bacteriophage T7 RNA polymerase can influence the phage growth [15]. However, the phage T7 genome encodes 56 genes, from which 56! ( = 10^74^) linear gene order permutations could be defined, so only a vanishingly small fraction of the total genome-design space could be examined by wet-lab experiments or computer simulations.

**Figure 2:**
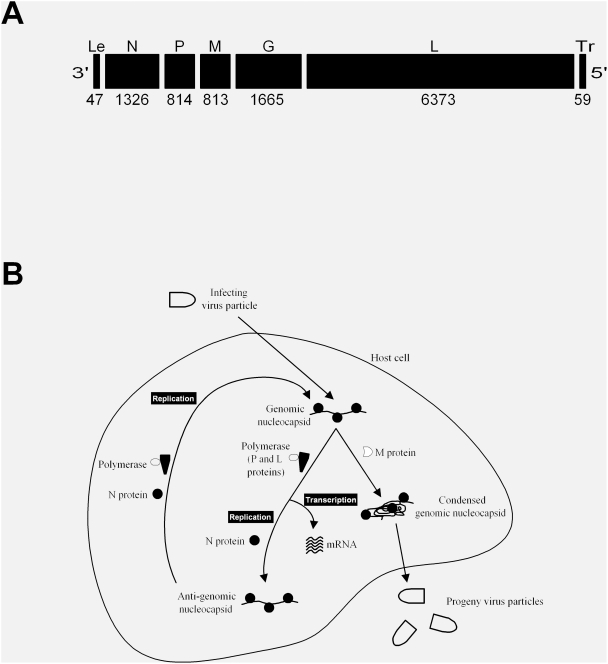
Overview of vesicular stomatitis virus (VSV). (A) Genome structure of VSV. Each gene is labeled by its single-letter abbreviation and length in nucleotides. The leader region (Le) encodes the genomic promoter, and the trailer region (Tr) encodes the complementary sequence to the anti-genomic promoter. (B) Growth cycle of VSV. The viral genomic RNA is used as a template for transcription of viral mRNA, which are translated to produce viral proteins. Accumulation of viral proteins enables amplification of the viral genome through an anti-genomic intermediate. Viral genomes are condensed, packaged and released as viral progeny into the extracellular environment.

Here we consider a relatively simple prototype of the (–)ssRNA viruses, VSV, which has been widely studied and well characterized [3],[16]. As shown in Figure 2A, VSV encodes five genes (N, P, M, G, and L), and these genes define 120 gene-order permutations. The five VSV genes play well-established roles in the growth of VSV, as summarized in Figure 2B. Very briefly, the entering negative-sense RNA genome is transcribed from its 3′ single promoter (called leader region (Le)) by the virion-associated VSV polymerase (proteins P and L). A controlled attenuation of transcription occurs in each intergenetic region, where a fraction of elongating polymerases are released from the genomic templates, producing mRNA levels that progressively decrease from N to L. Specifically, at the Le-N, N-P, P-M, M-G, and G-L junctions 0, 25, 25, 25, and 95 percent of polymerases entering the junction are respectively released from the templates without transcribing any downstream genes [3],[11],[17]. Hence, the relative expression level of any gene depends on its position within the genome; moving genes toward the 3′ or 5′ end of the genome respectively increases or reduces their level of expression. As N protein accumulates, it associates with nascent viral RNAs, creating an RNA-protein (or “nucleocapsid”) complex that enables the elongating polymerase to bypass transcription attenuation signals at intergenic regions, causing a switch from transcription to genome replication. Further, as M proteins accumulate, they associate with and condense the genomic nucleocapsid, diverting it away from transcription and replication processes, while directing it toward the formation of progeny virus particles. Finally, particle budding from the cellular membrane incorporates protein G (not shown) into the surface of progeny viruses. Unlike viral transcription and replication, the synthesis of viral proteins relies mainly on host translation resources, whose availability can vary depending on the host cell type and the extent to which they are susceptible to infection-mediated inhibition of protein synthesis.

In previous work we developed and employed a mathematical model to simulate and analyze the life cycle of VSV [13]. The model accounted for the core regulatory mechanisms of VSV and incorporated available quantitative knowledge on interactions among viral and cellular components during infection. Model predictions for the growth dynamics of several gene-rearranged VSV variants qualitatively matched the experimentally observed growth ranking and gene expression patterns of the variants [13]. These results suggest that the model might be useful for gaining insights into the growth of other gene-permuted variants. Advances in reverse genetics systems and synthetic biology approaches have facilitated the construction of several genome-engineered virus mutants [18], but the generation of 120 gene-order permuted variants and experimental comparison of their life cycles remains a daunting task. Instead, we employed our mathematical model here to simulate and study how all gene-order permutations of the VSV genome would be predicted to influence its growth.

## Results/Discussion

### Dynamics of Growth of 120 Gene-Permuted VSV Variants in BHK Cells

**Figure 3:**
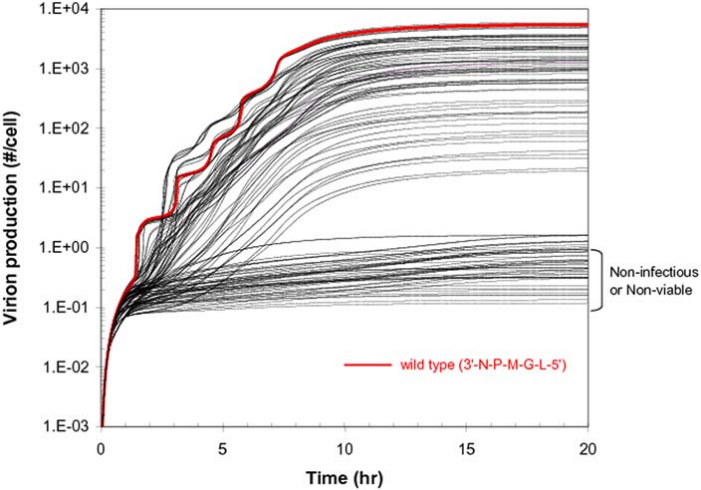
Simulated growth of all 120 gene-order permutations of VSV in the PRESENCE of transcriptional attenuation. Wild-type behavior is shown in red.

VSV has five genes in its genome in the order of 3′-N-P-M-G-L-5′. We generated *in silico* 119 all possible gene-permuted VSV mutants by keeping the wild type extents of transcriptional attenuation for the first to the fifth between-gene junctions (e.g., 0%, 25%, 25%, 25%, and 95%, respectively). Using our model we predicted the dynamics of their growth in baby hamster kidney (BHK) cells, and then we compared the dynamics with that of wild type virus (Figure 3). Here, the growth of wild type is the result of previous fitting of our model to experimental data [13]. In addition, parameters in the model were also constrained so simulated variants would satisfy the experimentally observed growth ranking of five mutants having gene orders 3′-N-n-n-n-L-5′, drawing n from P, M, and G [13]. Due to the existing attenuation mechanism, the gene-order shuffling yielded a large variation in the production of progeny virus particles. Depending on the gene order viable VSV variants produced from 1 to 6,000 progeny particles in an infected BHK cell (Figure 3). However, forty percent of the variants could not produce any progeny at all. Virion assembly started at around two hours post infection for wild type [16], but the timing was significantly retarded for most mutants (Figure 3). Although some variants showed faster growth patterns in the early infection stage between 2.5 and 5.5 hours post infection, the wild type virus overall grew better than most other variants (Figure 3). Only two mutants having the gene orders 3′-N-M-P-G-L-5′ and 3′-N-M-G-P-L-5′ produced more progeny particles than wild type.

### Effects of Gene Location on Viral Growth

**Figure 4:**
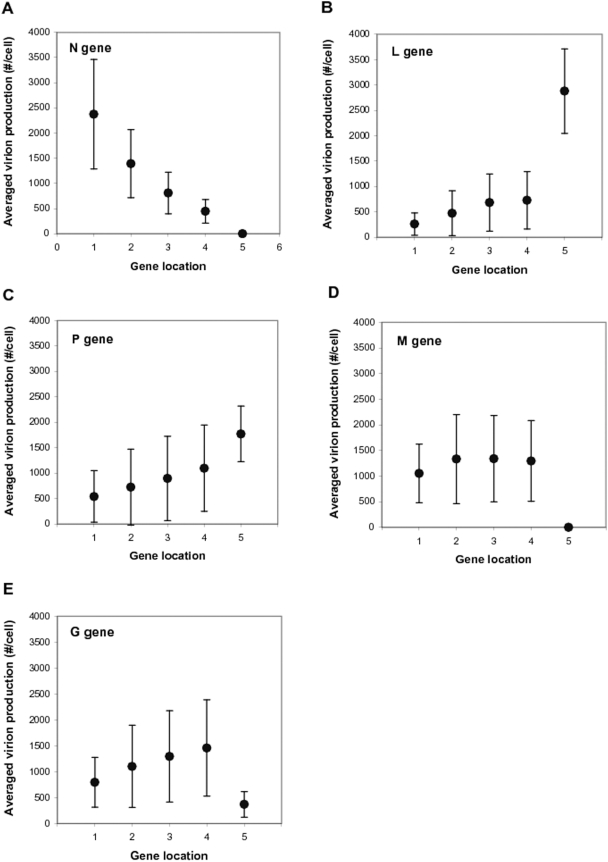
Effects of gene location on virus growth in the PRESENCE of transcriptional attenuation. Simulated yields from all 120 gene-order variants were grouped to show how the location of a specific gene impacts virus production. For each variant group, the mean virion production in BHK cells (filled circles) and its standard deviation (bars) is shown. There are a total of 25 variant groups (5 genes×5 gene locations), and each group has 24 virus variants.

To correlate the genome organization of each variant with its fitness, we first divided the 120 variants into five 24-variant groups, where all members of a group had a specific gene at a specific location. For example, all members of the N1 group have gene N in position 1, and the other positions are defined by the remaining 24 permutations of the four remaining genes. We then calculated the mean and standard deviation of the progeny virion production of the 24 variants in each group (Figure 4). Our analysis showed that for better viral growth, N gene, whose product is needed in a large quantity for genome encapsidation [2], should be located toward the 3′ promoter of the genome (Figure 4A), while L gene, whose product is needed in a low quantity for transcription and replication reactions, should be located toward the last position at the 5′ end of the genome (Figure 4B). Specifically, the variants of the L5 group grew much better than the variants of the other four groups (L1∼L4) (Figure 4B), highlighting the importance of minimal expression of L protein for viral growth. This model prediction is consistent with the experimental results that N-gene rearranged VSV variants grow better as N gene is located on earlier positions [19] and overexpression of L protein inhibits the virus growth [20]. In general, structural proteins are in a greater demand than enzymatic proteins during the viral infection cycle. However, the large variations in the virion productions of the N1 and L5 groups (Figure 4A and 4B) suggested that neither the assignment of N gene to the first genome position nor the assignment of L gene to the last position is a sufficient condition for optimal virus growth. The virion production gradually drops as P gene is moved toward 3′-proximal positions (Figure 4C). This is tightly coupled with the low composition stoichiometries of P protein in a VSV particle (Table 1) and in a polymerase complex with L protein. Moving P gene to earlier 3′-proximal genome positions will also reduces the expression of other genes whose products are needed in larger amounts. Due to the high composition stoichiometries of N, M and G proteins in a VSV particle (Table 1), when one of the three genes is located on the last genome position, the viral growth was severely reduced (Figure 4A, 4D, and 4E). The roles of M protein in condensing the genomic nucleocapsids and inhibiting host transcription additionally require a minimum level of M expression, putting an additional constraint that gene M avoid the last position. However, the location of M gene at any of the other fours positions did not strongly affect the viral growth (Figure 4D).

**Table 1:** Protein composition of VSV particle [23].

Proteins	Copies per virion
N	1258
P	466
M	1826
G	1205
L	50

Our analysis of 120,414 ranking vectors from the predicted growths of the 120 variants led to a more quantitative and systematic understanding of the effects of genome organization on the viral growth. First, the averaged ranking vector, [N, P, M, G, L]_BHK_ = [1.87, 3.57, 2.57, 2.90, 4.09] re-emphasizes that for better virus growth N and L genes need to be located on the first and the last genome positions, respectively. The large difference between the rankings of N and L genes (4.09−1.87 = 2.22) quantifies how important such a genome position separation of the two genes is for the viral growth. The voting results from progeny virions indicate that the gene order, 3′-N-M-G-P-L-5′, is the most common form to which genome organizations of many progeny virions match more closely than to any other gene order. This further implies that moderate alterations from this identified gene order would likely less perturb virion production compared to alterations from any other gene order. Therefore, the gene order, 3′-N-M-G-P-L-5′, can be considered a robust form of genome organization. Our second-order analysis using a *Pairs* matrix, where a component (*i*, *j*) quantifies to what extent gene *i* (listed in the first column) is preferred to gene *j* (listed in the first row) for an earlier genome position (Table 2), reinforced our previous results: preferences for genes in early positions start with N, and are followed by M, G, P, and L.

**Table 2:** Second-order ranking data analysis for BHK cells (with attenuation).

	N	P	M	G	L
N		0.819	0.676	0.733	0.906
P	0.181		0.283	0.372	0.599
M	0.324	0.717		0.565	0.820
G	0.267	0.628	0.435		0.770
L	0.094	0.401	0.180	0.230	

The first column and the first row list component i and j for *Pairs*, respectively.

### Some VSV Variants Can Also Grow Better Than Wild Type in Another Cell Type

**Figure 5:**
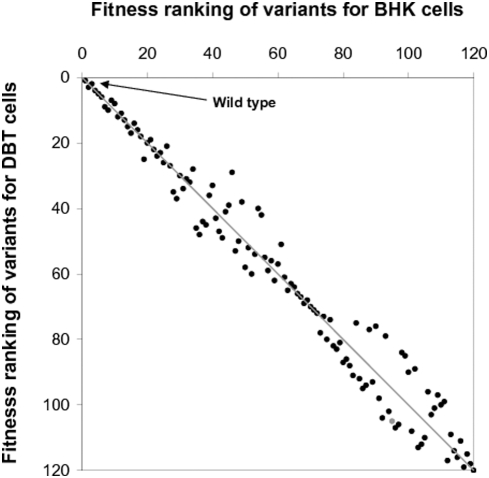
Effects of host cell on fitness rankings of gene-order variants. To establish the rankings the productivity of each VSV variant is compared with all other variants based on their simulated growth, in the presence of transcriptional control, in BHK and DBT cells. The dual ranking of each variant is represented by a single point in the figure. The most productive virus, which has fitness rank 1 on BHK and DBT cells, appears as a point in the upper-most left corner.

Experiments showed that several gene-shuffled VSV mutants can grow like or better than wild type [11]. Those mutants had the gene orders 3′-N-M-P-G-L-5′ and 3′-N-M-G-P-L-5′. Our previous model fitting results also suggested that increasing the VSV growth rate by gene rearrangement is feasible based on the given VSV regulatory circuit [13]. From the conventional hypothesis that wild type has the most evolved form of genome organization, results of others' study and our simulations raise clear questions: Why is wild type not the fittest? Could the fitter variants still grow better than wild type in many different cell types? Can any other variants grow better than wild type in some cell types? How does gene order systematically affect VSV growth in different cell types? To obtain some clues we compared *in silico* the growth of the 120 variants in BHK cells with their growth in delayed brain tumor (DBT) cells. Our previous model fitting to the experimental growth of wild type VSV in DBT cells suggested that resources of BHK cells for translation were 6 fold richer but 1.4 fold less stable compared to those of DBT cells [13]. Because host factors are mainly involved in VSV translation rather than in transcription and replication [16], such features relevant to translation would be the most representative basis to distinguish each host cell type as a different supporting environment for VSV growth. Out of the 120 gene-shuffled variants, the wild type grows second in DBT cells (third in BHK cells), which suggests that the wild type gene order might be a slightly sub-optimal or near-optimal product of natural selection (Figure 5). However, the existence of a mutant (3′-N-M-P-G-L-5′) whose simulated infection is more productive than wild type in different hosts, raises questions on the origin of the wild-type genome organization.

The virion production ranking of the VSV variants for BHK cells is roughly maintained for DBT cells as shown by the upper-left to lower-right diagonal pattern of variant growth rankings (Figure 5). Specifically, the high fitness rankers (≤18^th^) for BHK cells also grow better than other variants in DBT cells. The maintained fitness benefits from these specific gene orders even in the significantly different environments imply that such benefits likely arise from enhanced efficiencies of intrinsic viral regulatory mechanisms by the specific genome organizations, rather than from altered virus-host interaction patterns. However, mutants ranked between 19^th^ and 62^th^, 73^th^ and 120^th^ showed moderate variations in their relative virion productions depending on the host cell type (Figure 5). This indicates that the extent of virus fitness change by its gene order permutation also depends on the availability and stability of host factors that vary over host cell types.

In addition, we also used two types of metrics, Averaged rankings and *Pairs*, to compare the importance of gene order in the two cell types. First, the averaged ranking of each gene indicates the position in the genome where the gene needs to be located for productive viral growth. Second, as the component *K_ij_* in *Pairs*, corresponding to gene *i* and gene *j*, is closer to 1 and 0, gene *i* is more and less preferable, respectively, for an earlier genome position compared to gene *j* (See the Method section). The larger ranking difference in DBT cells between N and L genes (4.23−1.59 = 2.64) in the averaged ranking vector, [N, P, M, G, L]_DBT_ = [1.59, 3.48, 2.72, 2.97, 4.23], compared to the case of BHK cells (2.22), showed that locating N and L genes to the first and the last genome positions is more important for viral growth in DBT cells. However, the smaller standard deviation (SD) of the rankings of the three other genes, M, P, and G (SD of 2.72, 3.48, and 2.97 = 0.39 (DBT) vs. 0.51 (BHK)) revealed reduced importance of their genome positions compared to the case of BHK cells. The increased difference between rankings of N and L genes for DBT cells compared to the case of BHK cells (2.64−2.22 = 0.42) is equivalent to the standard deviations of the rankings of other three genes (0.39 and 0.51). This indicates that such host effects are at a level equivalent to the effect of relative positions of M, P, and G genes on viral growth. In addition, the values of the Pairs *K_ij_* for i = N and L are closer to 1 and 0, respectively, than the case of BHK cells, and the values of the Pairs *K_ij_* for i = P, M, or G and j = P, M, or G (Table 3) are all closer to 0.5 (Table 2), highlighting increased importance of the genome positions of N and L genes. Therefore, relative genome positions of genes affect the viral growth to a different extent depending on the type of host cells.

**Table 3:** Second-order ranking data analysis for DBT cells (with attenuation).

	N	P	M	G	L
N		0.870	0.784	0.816	0.936
P	0.130		0.325	0.402	0.662
M	0.216	0.675		0.547	0.839
G	0.184	0.598	0.453		0.796
L	0.064	0.338	0.161	0.204	

The first column and the first row list component i and j for *Pairs*, respectively.

### Growth of 120 Gene-Permuted Variants in the Absence of Transcriptional Attenuation

The role of gene order in controlling the relative protein expression level of (–)ssRNA viruses is tightly linked to the partial transcription termination mechanism by which genes more proximal to the 3′ promoter region are favored for transcription. Such an attenuation mechanism would be obtained through evolution to satisfy different degrees of need of each protein during viral infection cycles. If no attenuation mechanism is available, would the wild-type gene order be more productive than all other gene-order mutants? If so, one might speculate that the wild type gene order was fixed before VSV adopted the current attenuation mechanism.

**Figure 6:**
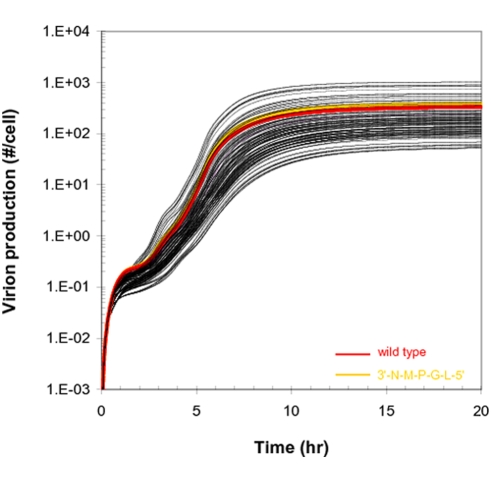
Simulated growth of all 120 gene-order permutations of VSV in the ABSENCE of transcriptional attenuation. Highlighted in different colors are wild-type (red) and a variant with gene order 3′-N-M-P-G-L-5′ (yellow).

To test this hypothesis *in silico*, we predicted the growth of the 120 variants again in the absence of an attenuation mechanism. If a polymerase starts transcription from the 3′ promoter region in the absence of attenuation, then it will move through the whole genome, ultimately synthesizing all the five gene transcripts without being released from the genomic template. Even without any attenuation mechanism, all the gene-shuffled variants can produce progeny particles in BHK cells (Figure 6), a sharp contrast to the case of the wild type attenuation showing that 40 percent of variants produced no progeny (Figure 3). However, the variation of virion production by gene-order shuffling is just 20 fold (Figure 6), which is significantly smaller compared to that of the wild type attenuation case (more than three log variation as shown in Figure 3). Interestingly, even without an attenuation mechanism, some gene orders are more advantageous than others for viral growth. Due to the time required for polymerase to move from one end of the genome to the other end, there are still spatial polymerase concentration gradients on the genomic templates as the intracellular concentrations of L and P proteins fluctuate during infection. These spatial concentration gradients are smaller in the absence of active attenuation, but they are sufficient to generate differences in the levels of different viral mRNAs over time in a gene-order dependent manner. Thus, some VSV variants are fitter than others, even in the absence of attenuation.

**Figure 7:**
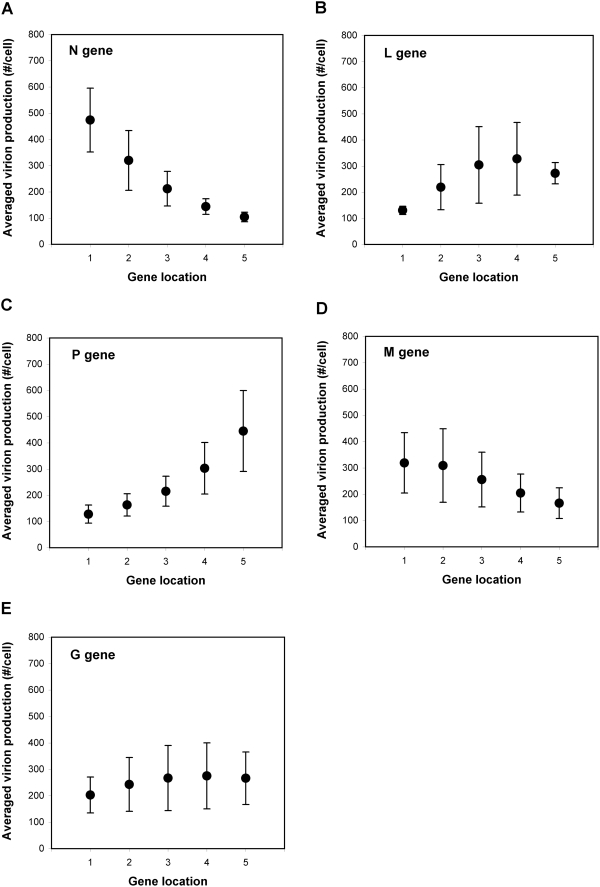
Effects of gene location on virus growth in the ABSENCE of transcriptional attenuation. The growth productivities for all 120 gene-order variants, simulated on BHK cells in the absence of transcriptional control, are grouped to show how the location of a specific gene impacts virus production.

In the absence of regulated gene expression our variant grouping analysis also showed significantly reduced importance of genome positions of each gene (Figure 7). For example, the N1 group produces only 4.5 fold more virion particles on the average than the N5 group (Figure 7A), a much smaller difference compared to 2900 fold in the presence of the wild type attenuation (Figure 4A). Interestingly, the L3 and L4 groups can produce more virion particles than the L5 group to which wild type virus belongs (Figure 7B). This suggests that in the absence of attenuation L protein expression does not have to be minimized for viral growth. Further, the N5 and M5 groups showed significant levels of virion production, which is also in contrast to the case of the wild type attenuation (Figure 4A and 4D versus Figure 7A and 7D). The averaged ranking vector, [N, P, M, G, L]_BHK-noATT_ = [2.27, 3.62, 2.67, 3.13, 3.31], highlights a reduced importance of gene order. For example, the ranking gap between the first and the last rankers is only 1.35 ( = 3.62−2.27), significantly reduced from 2.22 in the case of attenuation. Despite the reduced effect of gene order, the first genome position is still strongly preferred by N gene for viral growth.

**Figure 8:**
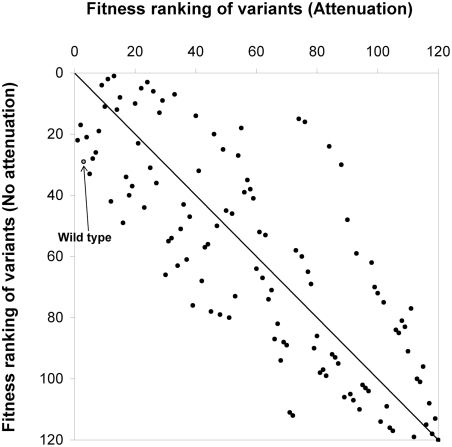
Effects of transcriptional regulation on fitness rankings of gene-order variants. The intracellular growth of virus from BHK cells infected by each gene-order variant was simulated in the presence and absence of transcriptional attenuation. Virus yields from these simulations were used to establish rankings for each variant.

The fitter VSV variants in the presence of the wild-type attenuation mechanism tend also to be fitter in the absence of attenuation, as indicated by the upper-left to lower-right diagonal pattern of growth rankings (Figure 8). However, large deviations from the diagonal indicate a lack of strong correlation. Without an attenuation mechanism, wild type still grows well in BHK cells, but just within the top 24 percent of the 120 variants, compared to its ranking in the top 2 percent in the presence of the wild type attenuation mechanism (Figures 5 and 8). In addition, the mutant having the gene order 3′-N-M-P-G-L-5′, which was a fitter compared to wild type in the presence of attenuation, still grows better than wild type in BHK cells. The large number of mutants that are predicted to grow better than wild type in the absence of attenuation has evolutionary implications. Specifically, if natural selection was critical for the fixation and conservation of the wild-type gene order, then the attenuation mechanism should have preceded or co-evolved with gene order.

### Evolution of VSV Gene Order

We have shown that the wild type ranks highly under the attenuation mechanism (Figures 3 and 5). However, the existence of a few mutants that grow like wild type or better could not be clearly explained by our simulations. Several factors may be relevant. BHK and DBT cells may significantly differ from the cell types that VSV infects in nature. Our model may well still lack information on unknown functions of viral proteins or their interactions with cellular components that affect growth. We finally suggest another reason why the sub-optimal genome organization for viral growth was fixed from VSV evolution. Instead of gene-rearrangement requiring a series of complicated recombination steps, there could have been an alternative mechanism to increase the viral fitness by fine-tuning the relative gene expression level. In this setting transcriptional attenuation would be a plausible mechanism. We have already shown the importance of the attenuation mechanism for viral growth under limited host resources. The maximum and the average burst sizes of the 120 variants for BHK cells were increased by 5.8 and 4.0 fold, respectively, in the presence of the wild type attenuation mechanism compared to the case of no attenuation (Figures 3 and 6). In particular, the growth of wild type was increased by 16.3 fold in the presence of attenuation. Further, our simulations showed that changes of extents of attenuation at gene junctions can increase the virion production of wild type in BHK cells by 24 percent (unpublished data). In addition, depending upon the attenuation pattern virion production from the wild type gene order could vary by 6700 fold (unpublished data). From these predicted large variations of growth phenotype by mutations to the attenuation mechanism, we conjecture that perturbations of the degrees of attenuation at each intergenic region by point mutations could have provided a means for VSV to more readily adapt to new host environments than by re-ordering the wild type genome. This idea is in part supported by experimental observation showing that a few point mutations at an intergenic region of VSV could cause transcriptional attenuation to span from 5 to 98 percent [21]. The control of relative level of viral transcripts is the central mode of regulation during VSV infection cycle. We suggest that VSV has obtained a near-optimal transcription control by co-evolution of its gene order and intergenic sequences instead of relying only upon gene-order optimization for growth.

## Methods

### Model Simulations

To consider in detail the transcription attenuation mechanism of VSV, we modeled the spatial and temporal changes of polymerases distributed along the viral genome templates during our simulations of the viral infection cycle [13]. We first partitioned the genomic templates into multiple segments, then estimated the polymerase flux into each segment at each time point post infection, and finally correlated the polymerase occupancy on a fixed number of segments corresponding to each gene with its temporal transcription level [13]. By changing the gene scanning order of polymerase *in silico*, our model could be easily extended to predict the growth dynamics of gene-shuffled VSV variants. While the gene order of each variant affects its transcription pattern, we assumed the intrinsic interactions among encoded viral proteins and RNAs to be conserved among all variants.

### Statistical Ranking Data Analysis

Using our model we simulated the cell infections by each of the 120 gene-shuffled VSV variants and determined the resulting yield of virus progeny. For example, two virus variants having the gene orders 3′-G-L-M-N-P-5′ and 3′-L-M-G-P-N-5′ produced 645 and 1 virion particles in an infected BHK cell, respectively. Based on their virion production, they were ranked as 48^th^ and 80^th^, respectively. By grouping the 120 VSV variants based on the genome position of a specific gene and comparing the averaged progeny production of each group, we quantified how increasing or decreasing the relative expression level of a single gene affects the virus growth. For example, based on the location of N gene five groups can be defined (e.g., 3′-N-n-n-n-n-5′ (N1), 3′-n-N-n-n-n-5′ (N2), through (N5), where n is either P, M, G, or L). Each group consists of 24 virus variants that contribute to the calculation of the average (mean) and standard deviation of virus production for the group.

To better understand how relative gene order impacts progeny production we viewed the simulated virus growth as voting results. The 120 VSV variants produced a total of 120,414 virion particles in individually infected BHK cells. Now we assume that each virion particle as a voter ranks five different candidates (N, P, M, G, and L). For example, 645 virion particles (having the gene order, 3′-G-L-M-N-P-5′) choose G as the first ranker, L as the second, and so on. From this voting result we can construct 645 ranking vectors for these 645 virion voters (y_1_, y_2_, … y_645_ = [4],[5],[3],[1],[2] ^T^). In each ranking vector we put the rankings of N, P, M, G, and L, first to fifth, respectively. In this manner we generated 120,414 ranking vectors for the total 120,414 virion particles. Two metrics calculated from such ranking vectors systematically quantified the impacts of the location of each gene as well as interactions among locations of different genes.

*Averaged ranking* [22]



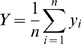
(1)where *y_i_* is a ranking vector, *n* is the number of total virion particles as voters, and *Y* is the averaged ranking vector for the virion voter population. Depending on the type of host cell that interacts with viruses and has a certain level of resources for biosynthesis, *n* will vary.

The averaged ranking can inform where a single gene needs to be located within the genome for productive viral growth. For example, from *Y* = [3.21, 1.32, 4.33, 2.21, 4.67]^T^ we can infer that for viral growth N, P, M, G and L genes need to be located at the third, first, fourth, second, and fifth genome positions, respectively.

*Pairs* [22]



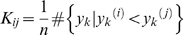
(2)where if the ranking of component *i* is higher than the ranking of component *j* for the ranking vector k, then count one. After the counting process for all the ranking vectors, the obtained numbers are summed (#) and then divided by the number of total virion voters (*n*). *K* is the *Pairs* matrix (5×5 for our case) that shows which gene should be followed by which gene for productive viral growth. As a numerical example, we assume that we have three ranking vectors (or voting results), [2],[4],[1],[3],[5] ^T^, [2],[4],[5],[1],[3] ^T^ and [3],[1],[5],[4],[2] ^T^, and we consider component 1 and 2. For *K_12_* we obtain 2/3 by counting 2 from the first two ranking vectors and then by dividing it by 3 ( = n). As the number corresponding to gene *i* and gene *j* (*K_ij_*) is closer to one, gene *i* is more preferable for an earlier genome position compared to gene *j*. In contrast, as the number is closer to 0, then gene *i* is less preferable to gene *j*. If the number is close to 0.5, then the relative genome positions of gene *i* and *j* are not important for the virus growth.
